# Population size interacts with reproductive longevity to shape the germline mutation rate

**DOI:** 10.1073/pnas.2423311122

**Published:** 2025-05-20

**Authors:** Luke Zhu, Annabel Beichman, Kelley Harris

**Affiliations:** ^a^Department of Bioengineering, University of Washington, Seattle, WA 98195; ^b^Department of Genome Sciences, University of Washington, Seattle, WA 98195; ^c^Computational Biology Division, Fred Hutchinson Cancer Center, Seattle, WA 98109

**Keywords:** mutation rate, nearly neutral theory, generation time, longevity, germline

## Abstract

Mutation rates vary across the tree of life by many orders of magnitude, with fewer mutations occurring each generation in species that reproduce quickly and maintain large effective population sizes. A compelling explanation is that large effective population sizes facilitate selection against weakly deleterious “mutator alleles” such as variants that modulate cell division or interfere with the molecular efficacy of DNA repair. However, while the fidelity of a single cell division largely determines microorganisms’ mutation rates, the relationship of the mutation rate to the molecular determinants of DNA damage and repair is more complex in multicellular species with long generation times. Since long generations leave more time for mutations to accrue each generation, we posit that a long generation time likely amplifies the fitness consequences of any damage agent or DNA repair defect that creates extra mutations in the spermatogonia or oocytes. This leads to the counterintuitive prediction that the species with the highest germline mutation rates per generation are also the species with most effective mechanisms for avoiding and repairing mutations in their reproductive cells. Consistent with this, we show that mutation rates in the reproductive cells are inversely correlated with generation time; in contrast, the number of germline mutations that occur during prepuberty development trends weakly upward as generation time increases. Our results parallel recent findings that the longest-lived species have the lowest mutation rates in adult somatic tissues, potentially due to selection to keep the lifetime mutation load below a harmful threshold.

## Significance

All cells accumulate mutations throughout life, despite an intricate network of molecular mechanisms that are designed to safeguard DNA from such alteration. Recent studies of somatic mutations have found that species with longer lifespans appear to accumulate mutations more slowly in certain somatic tissues, suggesting that longevity intensifies the selective pressure for effective DNA repair and other mechanisms of mutation avoidance. Here, we show that the same dynamic appears to affect the germline: Although longer-lived species transmit more mutations to their offspring, this trend is dampened by selection to minimize mutation rates in the reproductive cells of long-lived organisms.

Germline mutation rates vary by orders of magnitude across the tree of life and ultimately limit the adaptability and the complexity of each species (1–5). Low mutation rates may limit the rate of adaptation to new challenges (6–8), while high mutation rates may limit the ability of a well-adapted population to maintain its fitness and dominance (9, 10). Maintenance of a low mutation rate also incurs an energetic cost, requiring investment of resources and genomic real estate in DNA repair machinery and other mutation-avoiding systems (11–14). As organisms get more complex, the possible consequences of a high mutation rate get more complex as well, leading to confusion and debate about which evolutionary forces ultimately shape this important parameter (15–17).

One widely cited model, the drift barrier hypothesis, posits that mutation rate variation is largely driven by differences in effective population size that modulate the efficacy of selection against weakly deleterious alleles (5, 18–20). A “mutator allele” that raises the germline mutation rate is likely to be deleterious given that harmful mutations outnumber beneficial mutations, but since most mutations are neutral or only weakly harmful, a modest increase in the mutation rate is only expected to decrease fitness by a small amount (21, 22). A corollary of the drift barrier hypothesis is that genetic drift likely limits the ability of DNA repair enzymes to function near their biophysical optima, since optimal functioning would require natural selection to weed out mutator alleles that cause very few additional germline mutations each generation and thus have nearly neutral fitness effects (23). As a result, different nearly neutral mutator alleles are likely to accumulate over time in each population and species, causing the molecular efficacy of each DNA repair enzyme to diverge across the tree of life (24, 25). Although there exists little direct data on the molecular efficacy of DNA repair and how it varies among species, the predictions of the drift-barrier hypothesis enjoy broad indirect support from mutation rate data, which are easier (though still expensive) to measure. Across the tree of life, population size is inversely correlated with the mutation rate per site per generation (26), and a similar correlation was recently measured using vertebrate mutation rate data alone (27).

In single-celled organisms, there is a fairly direct connection between DNA repair efficacy and mutation rate per generation (which is the same as the mutation rate per cell division). Single-celled organisms also exhibit substantial diversity in the architecture of DNA repair, ranging from the minimalist repair systems of some obligate symbionts [which have very high mutation rates (28)] to unique genomic proofreading mechanisms in ciliates such as *Paramecium*, which have some of the lowest mutation rates known to science (29–31). In contrast, multicellular eukaryotes have more standardized cellular housekeeping processes but varied, multistage life histories, with each generation involving multiple cell divisions as well as potentially mutagenic cell states associated with sex and embryonic development (32–34). This complexity muddies the relationship between the mutation rate per generation and the molecular efficacy of processes such as DNA proofreading and repair, thereby complicating the interpretation of the correlation between mutation rate and effective population size. When Bergeron et al. noted that effective population size was correlated with the per-generation mutation rate among vertebrates, they noted that a possible explanation for this correlation was covariation between effective population size and generation time: the typical age at which reproduction occurs (27). A strong positive correlation between generation time and the mutation rate per generation was previously inferred from phylogenetic substitution data, and the etiology of this pattern has been long debated (16, 17, 35). Within species, later reproduction increases the mutation rate independently of molecular DNA repair efficacy, and this parental age effect might also be responsible for some proportion of mutation rate variation between species (36, 37).

The effect of parental age on the human mutation rate has been well characterized thanks to the availability of thousands of mutation rate measurements from trios where the ages of the parents at the birth of the child are known (38). Similar (though smaller) trio datasets have also been generated for several nonhuman mammalian species, and all show the same qualitative pattern of increasing mutation rate per generation as a function of parental age (39–45). These data show evidence of significant mutation rate differences among species, and they also differ in estimates of how quickly the mutation rate per generation increases with the ages of the father and mother. However, the small sample sizes of most nonhuman mutation rate studies come with high degrees of statistical uncertainty, and some recent studies of mutation rates in primates and carnivores have argued that parental age effects in these species are not statistically distinguishable from each other (40, 44). Instead, they found that mutation rate measurements from several primate species, as well as the domestic cat, were consistent with a *reproductive longevity model* where most mutation rate divergence among species is driven by differences in the timing of puberty and reproduction. Other studies have found that mutation rates vary among human families (46, 47) and among dog breeds (45) even after conditioning upon the age of reproduction, but in humans, a high mutation rate appears to be associated with reduced fertility and higher all-cause mortality (48). If alleles that drive mutation rate variation within species have deleterious pleiotropic effects, they might not persist over long enough evolutionary time periods to create mutation rate differences between species.

Here, we study the etiology of vertebrate mutation rate variation by decomposing the germline mutation rate into its three main components: the number of mutations that accumulate each generation during embryonic and prepubescent development, the rate of mutations occurring in the spermatogonia and oocytes per year of adult reproductive life, and the length of time elapsed between puberty and reproduction. We show that the mutation rate in the spermatogonia and oocytes postpuberty is likely to be lowest in species with long generation times, implying that the species with the highest mutation rates per generation may paradoxically be the species with the most effective DNA repair and proofreading machinery. This scaling reverses the direction of one drift-barrier hypothesis prediction, implying that selection against certain mutator alleles will be most effective in species with long generation times, not in species with large effective population sizes that tend to have short generation times.

We test our predictions by estimating the three aforementioned parameters using published regressions of mutation rate against generation time in eight mammalian species. Consistent with our model, we find that generation time appears to be weakly positively correlated with the prepuberty mutation rate but significantly negatively correlated with the postpuberty mutation rate in spermatogonia and oocytes. This suggests that the rate of mutations accumulating before puberty conforms to predictions of the classic drift-barrier hypothesis, but postpuberty germ cell mutation rates are better described by a modified drift-barrier model where the strength of selection against mutators is intensified by late reproduction.

## Results

### Variation in the Rates of Germline Mutations Occurring During Different Life History Stages.

Let $\mu_{S}$ and $\mu_{O}$ denote the mutation rates per site per year occurring in the spermatogonia and primary oocytes of reproductively mature individuals. There are also additional germline mutations that occur in the early embryo, the primordial germ cell lineage, and the immature gamete precursors that exist prepuberty: We will refer to this cumulative “early” mutation load as $\mu_{E}$, measured in mutations per site per generation. In terms of these rate parameters, the age of puberty (P), and the ages of maternal and paternal reproduction ($A_{M}$ and $A_{P}$), the germline mutation rate μ per site per generation is

[1] $$\mu =\mu_{E}+(A_{P}-P)\cdot \mu_{S}+(A_{M}-P)\cdot \mu_{O}.$$

**Fig. 1.**
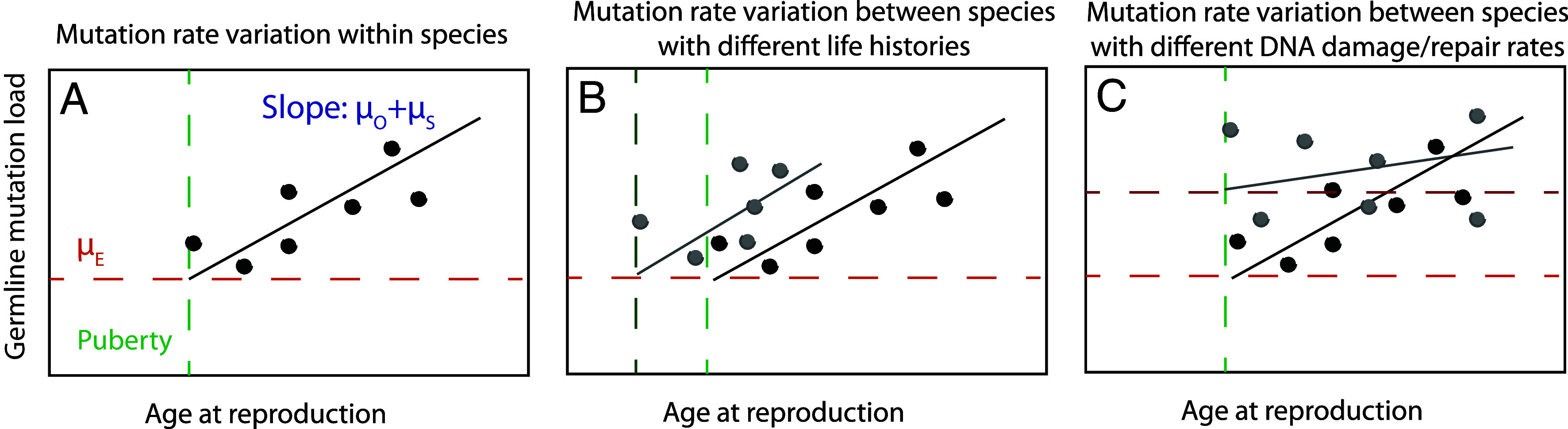
Models of germline mutation rate variation. (*A*) Within species, mutation rates vary as a function of age at reproduction. Each individual is expected to accumulate a prepuberty mutation load $\mu_{E}$ plus inherit additional mutations that have accumulated in their parents’ spermatogonia and oocytes at rate $\mu_{O}+\mu_{S}$ each year between puberty and conception. (*B*) Two species with different lifespans and/or different ages of puberty onset may have different distributions of mutation rates despite similar mutation parameters $\mu_{E}$ and $\mu_{O}+\mu_{S}$, as posited in (40, 44). (*C*) Two species with similar lifespans and similar ages of puberty onset might still have different mutation rates due to genetic differences that affect rates of DNA damage, repair, or proofreading. This type of mutation rate variation is driven by variation in the parameters $\mu_{E}$ and/or $\mu_{O}+\mu_{S}$.

The reproductive longevity model by Thomas, Wang, and colleagues posits that most mammalian mutation rate variation can be explained by variation in the age parameters $A_{P},A_{M}$, and P rather than the rate parameters $\mu_{S}$, $\mu_{O}$, and $\mu_{E}$ (40, 42, 44), as pictured in Fig. 1 *A* and *B*. This hypothesis was supported by early mutation rate measurements from owl monkeys and domestic cats, which appeared consistent with a model that combined human-trained values of $\mu_{E}$ and $\mu_{O}+\mu_{S}$ with species-appropriate ages of puberty and reproduction.

In theoretical contrast to this reproductive longevity model, Fig. 1*C* illustrates a model where mutation rate variation is primarily driven by variation in $\mu_{E}$ and $\mu_{O}+\mu_{S}$. At least some rate parameter variation is needed to explain mutation rate measurements from rhesus macaques and mice–compared to humans, macaques appear to have a lower $\mu_{E}$ (42) and mice have a fivefold higher spermatogonial mutation rate $\mu_{S}$ (49). We reanalyzed published mouse and human de novo mutation (DNM) data using a Poisson regression approach and found that both $\mu_{E}$ and $\mu_{O}+\mu_{S}$ had disjoint 95% CI between the two species (mouse $\mu_{E}=3.75\times 10^{-9}$, 95% CI $2.89\times 10^{-9}$ to $4.6\times 10^{-9}$; human $\mu_{E}=6.35\times 10^{-9}$, 95% CI $5.47\times 10^{-9}$ to $1.21\times 10^{-8}$; mouse $\mu_{O}+\mu_{S}=1.64\times 10^{-9}$, 95% CI $4.10\times 10^{-10}$ to $2.85\times 10^{-9}$; human $\mu_{O}+\mu_{S}=3.5\times 10^{-10}$, 95% CI $3.3\times 10^{-10}$ to $3.7\times 10^{-10}$).

To test for broader patterns of variation in $\mu_{E}$ and $\mu_{O}+\mu_{S}$, we searched the literature for parent/child trio data that would permit estimation of these parameters in additional species. We found appropriate data for five more primates plus one carnivore. We estimated $\mu_{E}$ and $\mu_{O}+\mu_{S}$ for each of these species using a Poisson regression of mutation count versus a weighted average of each trio’s paternal and maternal age. We weighted paternal age and maternal age in proportion to the relative rates of mutations occurring in spermatogonia and oocytes, as motivated by the following transformation of Eq. **1**:

[2] $$\begin{array}{c} \mu =\mu_{E}+(\mu_{O}+\mu_{S})\cdot [A_{P}\cdot \mu_{S}/(\mu_{O}+\mu_{S})\\ \;+A_{M}\cdot \mu_{O}/(\mu_{O}+\mu_{S})-P]. \end{array}$$

Gao et al. previously developed a maximum likelihood approach for estimating $\mu_{E}$, $\mu_{O}$, and $\mu_{S}$ from human trio data, which leverages information about the maternal and/or paternal haplotype phase of some mutations (50). Since partial mutation phase information is available for each species we included in this study, we were able to use the Gao et al. method to estimate $\mu_{S}/\left(\mu_{O}+\mu_{S}\right)$ for each species (*Methods*) and then compute the following weighted average parental displacement from the age of puberty.

[3] $$\bar{A}=A_{P}\cdot \mu_{S}/(\mu_{O}+\mu_{S})+A_{M}\cdot \mu_{O}/(\mu_{O}+\mu_{S})-P.$$

For each dataset, we also estimated the value of $\mu_{S}/\left(\mu_{O}+\mu_{S}\right)$ that maximized the correlation between mutation rate and the weighted parental age and found that these alternative estimates were close to our likelihood-based estimates (*Methods* and Dataset S4). In particular, the two methods agreed that $\mu_{S}>\mu_{O}$ for all species other than aye-aye and mouse. In the mouse, the likelihood approach found that $\mu_{S}>\mu_{O}$ but the correlation maximization approach lacked power because maternal and paternal ages were identical in every trio (49). In aye-aye, however, both methods agreed that $\mu_{O}>\mu_{S}$ in concordance with a previous report of unusual maternal mutation bias in this species (51, 52). Wang et al. previously reported that mutations were female-biased in the offspring of aye-aye mothers over age 15 y (52), and we infer that offspring mutation load is better correlated with maternal age than paternal age even when restricting the inference to offspring of mothers younger than 15 (Dataset S4).

After averaging together the parental ages, we estimated $\mu_{E}$ and $\mu_{O}+\mu_{S}$ using the following Poisson regression equation, letting U be the number of observed mutations per trio and L be the length in base pairs of the portion of the haploid genome that has sufficient read coverage to be accessible to DNM calling:

[4] $$U=\mu_{E}\cdot 2L+(\mu_{O}+\mu_{S})\cdot \bar{A}\cdot 2L.$$

Our estimates of $\mu_{E}$ and $\mu_{O}+\mu_{S}$, as well as each species’ average generation time *g* and age at first reproduction *P*, are compiled in Table 1.

**Table 1.** Regression-based estimates of embryo and gamete mutation rates

Species	Prepuberty mutation rate $\mu_{E}$ (muts/site/ generation)	Mutation rate $\mu_{O}+\mu_{S}$ in the germ cells after puberty (muts/site/year)	Age of puberty/ first reproduction *P* (years)	Generation time *g* (years)	Mutation rate μ (muts/site/ generation)
Human (38, 41)	6.6*e*−9	3.63*e*−10	13	30	1.24*e*−8
Chimpanzee (53)	1.07*e*−8	3.41*e*−10	14	25	1.44*e*−8
Olive baboon [Wu] (41)	4.59*e*−9	1.81*e*−10	5.4	10	5.42*e*−9
Olive baboon [Wang] (52)	5.14*e*−9	3.15*e*−10	5.4	10	6.59*e*−9
Rhesus macaque (42, 43)	3.78*e*−9	4.7*e*−10	3.5	8	5.9*e*−9
Owl monkey (40)	4.61*e*−9	6.27*e*−10	1	6.6	8.13*e*−9
Domestic cat (44)	5.7*e*−9	6.37*e*−10	0.5	3.8	7.8*e*−9
Mouse (49)	4.08*e*−9	1.51*e*−10	0.15	0.75	4.99*e*−9
Aye-aye [Wang] (52)	2.19*e*−10	1.27*e*−9	3	4	1.49*e*−9
Aye-aye [Versoza] (51)	5.83*e*−10	8.84*e*−10	3	4	1.43*e*−9

The generation times and ages at first reproduction in the table are drawn from the publications reporting each set of mutation rate data. See *SI Appendix* for a description of how these standardized rates were calculated from each study’s reported data.

We used nonparametric bootstrapping to assess the uncertainty associated with each pair of $\mu_{E}$ and $\mu_{O}+\mu_{S}$ parameter estimates (*SI Appendix*, Fig. S1). Within each set of bootstrapped estimates, we see an anticorrelation between $\mu_{E}$ and $\mu_{O}+\mu_{S}$ which is likely driven by the fact that an overestimation of a regression slope can partially compensate for an underestimation of the y-intercept. However, *SI Appendix*, Fig. S1 shows minimal overlap among the two-dimensional CI of parameter pairs from different datasets, indicating that the variation among species in mutation rate parameters exceeds the uncertainty associated with each species’ mutation rate parameter estimates.

### Longer Generation Times are Associated with Lower Mutation Rates Per Year in the Germ Cells.

**Fig. 2.**
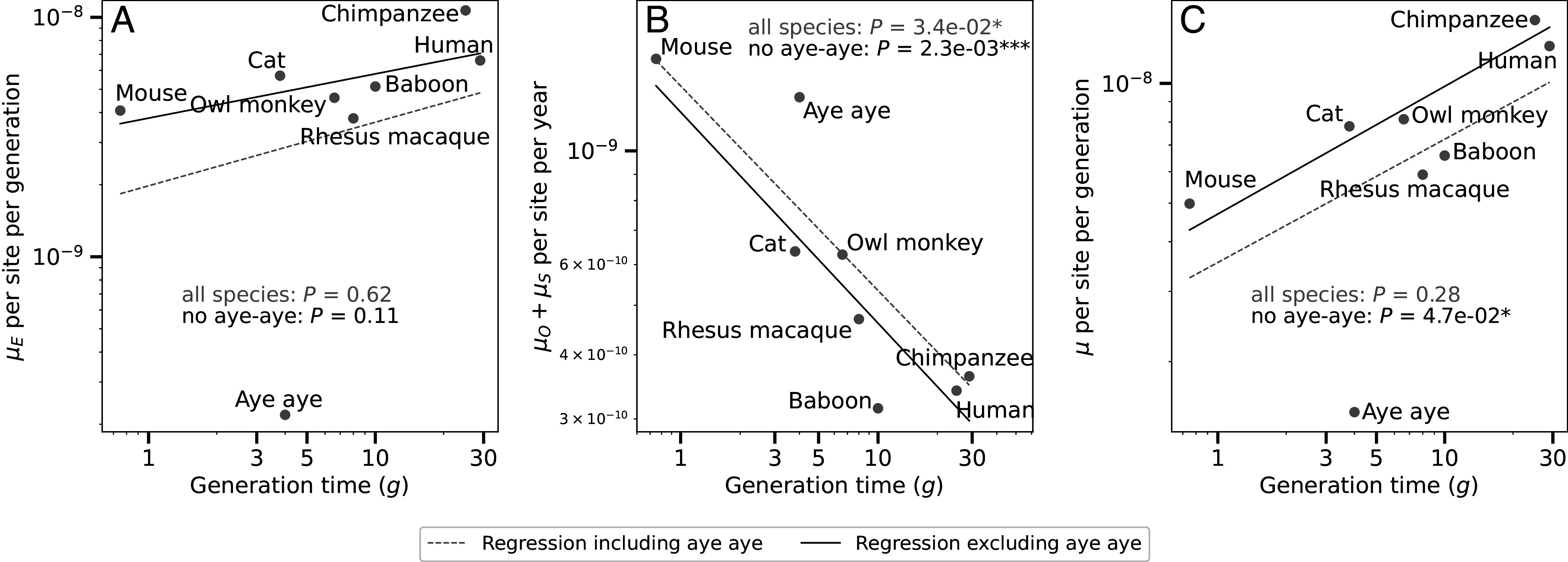
Variation among mammals in the rates of germline mutations occurring during early development versus in the post-puberty germ cells. (*A*) The prepuberty mutation rate $\mu_{E}$ trends upward with increasing generation time. (*B*) The mutation rate per year in the spermatogonia and oocytes postpuberty, $\mu_{O}+\mu_{S}$, is significantly negatively correlated with generation time. (*C*) The total germline mutation rate trends upward with generation time, echoing a finding that reached significance in previous analyses of larger datasets (26, 27).

We performed log–log–linear regressions of $\mu_{E}$, $\mu_{O}+\mu_{S}$, and $\mu =\mu_{E}+\left(g-P\right)\cdot \left(\mu_{O}+\mu_{S}\right)$ as functions of generation time (log–log linear regressions are more appropriate than natural scale regressions because the distributions of generation times and mutation rate estimates are closer to lognormal than normal, as shown in *SI Appendix*, Fig. S2). We performed all regressions using a phylogenetic least squares (PGLS) approach and found that $\mu_{E}$ trends upward with generation time, though the trend is not statistically significant (Fig. 2*A*, P>0.61). In contrast, the germ cell mutation rate $\mu_{O}+\mu_{S}$ is significantly inversely correlated with generation time (Fig. 2*B*, P<0.035). This result mirrors the inverse correlation between lifespan and the mutation rate per year of certain somatic stem cells, a pattern hypothesized to result from selective pressure to moderate cancer risk in long-lived species (54–56). As previously observed by Bergeron et al. (27) and Wang and Obbard (26), we find that the overall germline mutation rate trends upward with generation time, though this trend falls short of significance in our smaller dataset (Fig. 2*C*, P>0.28).

Since the aye-aye is an outlier in Fig. 2 *A* and *C*, we fit a second model to the data after excluding the aye-aye. Exclusion of the aye-aye increases the statistical significance of the negative correlation between $\mu_{O}+\mu_{S}$ and g to *P* < 2.3*e*−3, and increases the significance of the positive correlation between μ and g to *P* < 0.048. Moreover, we were able to determine that excluding aye-ayes results in a model that better describes mutation rate variation among a larger set of species (see section *A Variable Rate Reproductive Longevity Model Predicts Mutation Rate Variation Across the Full Range of Vertebrate Lifespans*.)

For several of the species represented in Table 1 and Fig. 2, multiple published DNM datasets were available. For each such species, we used a parental age regression with a categorical “dataset” variable to test for batch effects that might significantly affect the inferred values of $\mu_{E}$ and $\mu_{O}+\mu_{S}$ (Dataset S1). For rhesus macaque, we found no evidence of a batch effect and thus combined the available datasets. However, for baboon and aye-aye we did find that the model parameters depended significantly on the dataset being used. Moreover, these datasets contained some overlapping individuals, so for each species we picked only one dataset to use in our main analysis. Specifically, we utilized the baboon and aye-aye mutation calls generated by Wang et al. (52) because the same group called DNMs in many other species included here (specifically cat, owl monkey, and one rhesus macaque dataset). We made this choice to maximize pipeline consistency–we performed no benchmarking to assess whether the Wang et al. pipeline is more or less accurate than the pipelines used by other groups to infer DNMs from the same species. Moreover, when we repeated our regression using the alternative aye-aye and baboon datasets, we obtained the same qualitative results: a significant negative dependence of $\mu_{O}+\mu_{S}$ on generation time and a nonsignificant positive correlation between $\mu_{E}$ and generation time (*SI Appendix*, Fig. S3 *A*–*I* and Datasets S2 and S3).

The averaging together of paternal and maternal age introduces some additional uncertainty into our analysis. To measure how uncertainty in estimates of $\mu_{S}/\left(\mu_{O}+\mu_{S}\right)$ might be impacting our results, we repeated our inference of each species’ $\mu_{E}$ and $\mu_{O}+\mu_{S}$ values using a constant $\mu_{S}/\left(\mu_{O}+\mu_{S}\right)$ value of 0.75, which is the value estimated from thousands of human trios. This alternative inference scheme yielded very similar estimates of $\mu_{E}$ and $\mu_{O}+\mu_{S}$ for most species and did not change the qualitative relationships of these rate estimates to generation time as compared to our likelihood-based averaging scheme (*SI Appendix*, Fig. S3 *J*–*L* and Datasets S2 and S3). The only species whose $\mu_{E}$ and $\mu_{O}+\mu_{S}$ estimates appeared sensitive to the choice of parental age averaging scheme was the aye-aye, whose estimated $\mu_{S}/\left(\mu_{O}+\mu_{S}\right)$ value is the furthest from 0.75. Assuming that $\mu_{S}/\left(\mu_{O}+\mu_{S}\right)=0.75$ in aye-ayes results in a larger value of $\mu_{E}$ and makes this species appear much less of an outlier in *SI Appendix*, Fig. S3 *J*–*L* compared to Fig. 2.

### A Variable Rate Reproductive Longevity Model Predicts Mutation Rate Variation Across the Full Range of Vertebrate Lifespans.

We hypothesized that the relationships among generation time, $\mu_{E}$, and $\mu_{O}+\mu_{S}$ shown in Fig. 2 might be useful for predicting the mutation rates of species for which mutation rate measurements are unavailable, as well as testing whether excluding aye-ayes results in a model that better predicts broader patterns of mutation rate variation. To generate such predictions, we sought to approximate Eq. **1** as a relationship between *g* and μ rather than an equation depending on multiple demographic parameters (paternal age, maternal age, and the timing of puberty). We first substituted the generation time *g* for both the paternal age $A_{P}$ and the maternal age $A_{M}$, then utilized a large set of vertebrate demographic data to approximate the age of puberty as a function of *g* (57). We performed a linear regression of the age at first reproduction (*P*) against the average age at reproduction (*g*) and found that *P* is approximately equal to 0.42×*g* across 230 species with generation times ranging from 2 to 52 y (*r* = 0.87; *SI Appendix*, Fig. S4). Motivated by this, we further approximated Eq. **1** using the assumption that *p* = *P/g* is a constant across species such that g-P=g·(1-P/g)=g·(1-p) and

[5] $$\mu =\mu_{E}+g\cdot (1-p)\cdot (\mu_{S}+\mu_{O}).$$

We also let $\mu_{E,H}$, $\mu_{S,H}$, $\mu_{O,H}$, and $p_{H}$ be species-specific values of the corresponding parameters and substituted these values into (5) to predict mutation rate in the context of a strict reproductive longevity model that predicts the per-generation mutation rate μ based on parameters inferred from a single species such as humans:

[6] $$\mu =\mu_{E,H}+g\cdot (1-p)\cdot (\mu_{O,H}+\mu_{S,H}).$$

We then adapted Eq. **5** to formulate a model that allows $\mu_{E}$ and $\mu_{O}+\mu_{S}$ to vary as inferred from our meta-analysis in Fig. 2. To capture variation in $\mu_{E}$ as a function of generation time *g*, we let $\mu_{E}\left(g\right)$ denote the prepuberty mutation rate at a generation time of *g* and let α denote the slope relating $\log\;\mu_{E}\left(g\right)$ to logg. By these definitions,

[7] $$\text{log}\;\mu_{E}(g)=\text{log}\;\mu_{E}(1)+\alpha \;\text{log}\;g.$$

Exponentiating both sides of Eq. **7** yields:

[8] $$\mu_{E}(g)=\mu_{E}(1)\cdot g^{\alpha }.$$

To capture germ cell mutation rate variation in a similar way, we let β denote the slope of the regression relating $\log({\mu_{S}}^{\left(g\right)}+{\mu_{O}}^{\left(g\right)})$ to logg, such that

[9] $$\begin{array}{c} \text{log}(\mu_{S}(g)+\mu_{O}(g))=\text{log}\left[\mu_{S}(1)+\mu_{O}(1)\right]+\beta \;\text{log}\;g, \end{array}$$

and

[10] $$\mu_{S}(g)+\mu_{O}(g)=\left[\mu_{S}(1)+\mu_{O}(1)\right]\cdot g^{\beta }.$$

Substituting these values into Eq. **6** yields a prediction of the overall mutation rate:

[11] $$\mu =\mu_{E}(1)\cdot g^{\alpha }+g\cdot (1-p)\cdot \left[\mu_{S}(1)+\mu_{O}(1)\right]\cdot g^{\beta }.$$

This simplifies to

[12] $$\mu =\mu_{E}(1)\cdot g^{\alpha }+(1-p)\cdot \left[\mu_{S}(1)+\mu_{O}(1)\right]\cdot g^{\beta +1}.$$

**Fig. 3.**
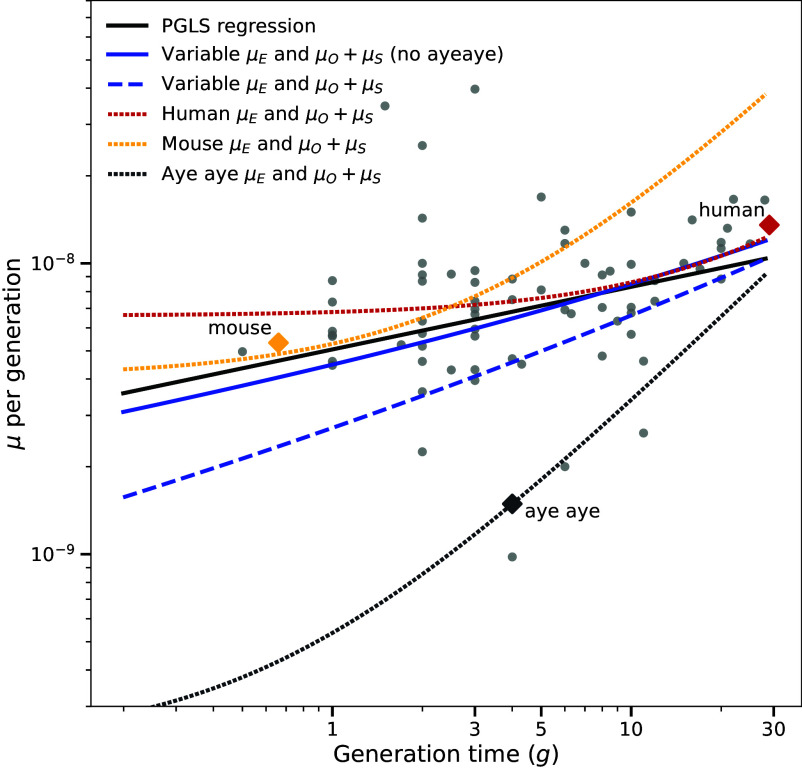
A comparison of constant-rate and variable-rate reproductive longevity models to the empirical correlation between mutation rate and generation time. A dashed line shows the PGLS regression (slope = 0.215, *P* < 6.7e−3) of mutation rate versus generation time in vertebrates from Wang and Obbard’s mutation rate meta-analysis (26). This is close to the prediction of the relaxed rate reproductive longevity model fit to the multispecies pedigree data (solid red line). The prediction of the fixed-rate reproductive longevity model with human parameters (orange dotted line) overestimates the mutation rates associated with short generation times, while the fixed-rate reproductive longevity model with mouse parameters (blue dotted line) overestimates mutation rates associated with long generation times. All fixed-rate reproductive longevity model predictions (aye-aye, mouse, and human) were generated with Eq. **6** using one generation time *g* for the paternal and maternal ages and species-specific values of the parameters $\mu_{E}$, $\mu_{S}$, $\mu_{O}$, and *P*.

Eqs. **6** and **12** make concrete predictions about how mutation rates should vary with generation time among vertebrates. We were able to compare these predictions to a large vertebrate mutation rate dataset that was recently compiled by Wang and Obbard (26). We used Eq. **12** to compare the Wang and Obbard data to two models pictured in Fig. 2: one fitted to all species and the other fitted to all species excluding aye-ayes. As shown in Fig. 3, only the model excluding aye-ayes is able to closely approximate the PGLS correlation between mutation rate and generation time in the Wang and Obbard data. In contrast, the human constant-rate reproductive longevity model (Eq. **6** with human-trained parameters) predicts higher mutation rates for species with short generation times. We also substituted mouse mutation rate parameters into (6) and found that the resulting model fits the mutation rates of short-generation-time vertebrates but overestimates the mutation rates of species with longer generation times. Compared to mice and humans, a model capturing the dependence of aye-aye mutation rate on generation time deviates more substantially from the dependence of mutation rate on generation time in other species.

One pertinent difference between human and mouse mutation rates is that mouse rates have been estimated from laboratory populations that have a long history of domestication. Bergeron et al. recently found that mutation rates per year were higher in domesticated species than wild species, possibly due to selection for rapid reproduction in captivity. However, we note that our mutation rate model with human parameters does not predict uniformly lower mutation rates per year than the corresponding model with mouse parameters– the mouse model only predicts lower mutation rates for generation times less than two years. In addition, the human and mouse models each predict a relatively constant mutation rate for generation times less than 1 y, which is the generation time range where these models predict that almost all germline mutations occur during prepuberty development.

### Long Lifespan Increases the Efficacy of Selection for a Low Mutation Rates in the Germ Cells.

The drift barrier hypothesis explains the inverse correlation between mutation rate and $N_{e}$ as a consequence of selection against weakly deleterious mutator alleles (19, 58). Mutator alleles might directly perturb DNA repair, proofreading, or cell division rates, or they might indirectly affect the mutation rate by perturbing a trait like metabolism. Species with larger effective population sizes are generally better able to eliminate weakly deleterious alleles, while species with small effective sizes are more likely to retain these alleles as a result of stronger genetic drift (59). This leads to the prediction that mutator alleles will be more prevalent in low-$N_{e}$ species, which also tend to have long generation times (60, 61). The germ cell mutation rate $\mu_{O}+\mu_{S}$ seems to contradict this prediction: We can extrapolate from Fig. 2*B* that species with the longest generation times and smallest effective population sizes are somehow the most effective at eliminating gamete mutator alleles. We can explain this contradiction by looking more closely at how the fitness effect of a mutator allele is calculated.

Let S(U) be the expected selection coefficient of a mutator allele that creates *U* additional mutations per genome per generation. Lynch previously estimated S(U) as follows (62): If each mutation has an expected fitness cost of E[s], then the expected per-generation fitness cost of the mutator allele is

[13] S(U)=UE[s].

In a population of effective size $N_{e}$, selection is predicted to eliminate mutations for which $S\left(U\right)>1/\left(2N_{e}\right)$. By this logic, natural selection should eliminate mutators whose per-generation mutation load *U* satisfies the inequality

[14] $$U>\frac{1/2N_{e}}{E[s]}=1/\left[2N_{e}E(s)\right].$$

**Fig. 4.**
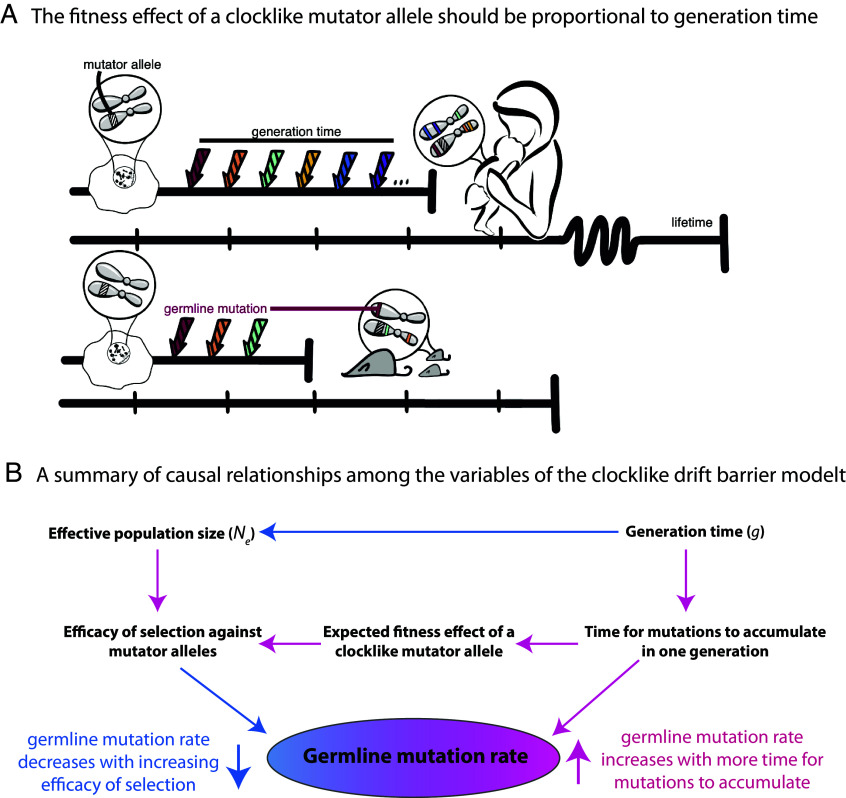
A model of germline mutation rate variation as a function of generation time, effective population size, and genetic variation that impacts the mutation rate measured per year. (*A*) Here, we compare the effects of identical molecular changes occurring in some human DNA repair gene as well as its mouse homolog. If these mutator alleles produce the same number of germline mutations per year, the human allele will produce a greater mutation burden per generation compared to the mouse allele, leading to a greater expected fitness cost and a larger negative selection coefficient in the long-generation-time species. *Figure credit: Natalie Telis.* (*B*) This diagram summarizes the multiple ways that generation time can affect the mutation rate, including its direct impact on the number of mutations that accumulate in a generation and its other impacts on the effective population size and the efficiency of natural selection. Pink arrows indicate positive correlations (an increase in the upstream variable causes an increase in the downstream variable), and blue arrows indicate negative correlations (an increase in the upstream variable causes a decrease in the downstream variable).

If we assume that *U*, $N_{e}$, *L*, and *E*[*s*] are essentially independent variables, then as $N_{e}$ gets larger, it will get progressively more difficult for a mutator to satisfy inequality (14) and thus the population should get more effective at purging away mutator alleles. A caveat is that it might not be reasonable to assume independence among *U*, $N_{e}$, and the generation time *g*. We can assume that *U* and *g* are independent when considering a mutator allele that modifies only $\mu_{E}$ while leaving $\mu_{O}+\mu_{S}$ unchanged, since such a mutator will create the same mutation load regardless of when the parents reproduce. However, for a mutator allele that alters $\mu_{O}+\mu_{S}$, creating extra mutations in the germ cells during each year of reproductive life, the total mutation load will scale proportional to *g*, as illustrated in Fig. 4*A*. This will shift the distribution of mutator allele fitness effects toward more deleterious values in species with long generation times, an idea that Lindsay et al. previously posited to explain why mice have higher per-year germline mutation rates than humans do (49). We will refer to such a modifier of $\mu_{O}+\mu_{S}$ as a “clocklike” mutator, in contrast to a “nonclocklike” mutator that modifies $\mu_{E}$ by a fixed amount each generation.

If a clocklike mutator increases the mutation load by *k* mutations per genome per year after puberty, the total number of extra mutations introduced per genome per generation will be kg(1-p). Substituting this mutation load for *U* in Eq. **13**, we predict that total fitness impact of the mutator each generation will be

[15] $$S\left[kg(1-p)\right]=kg(1-p)E(s).$$

Since $S\left[kg(1-p)\right]$ is proportional to the generation time *g*, we predict that selection against a clocklike mutator will get stronger as generation time increases, decreasing the mutation rate per year in the germ cells and explaining the trend in Fig. 2*B*. In order for the clocklike mutator to persist in the population, it must satisfy the familiar inequality $S(\left[kg(1-p)\right]>1/(2N_{e})$, which will only hold if

[16] $$\begin{array}{c} k>\frac{1/(2N_{e})}{g(1-p)E(s)}=1/\left[2gN_{e}(1-p)E\left(s\right)\right]). \end{array}$$

Inequality (14) defines a threshold of near-neutrality for modifiers of $\mu_{E}$, while inequality (16) defines a threshold of near-neutrality for modifiers of $\mu_{O}+\mu_{S}$. If we ignore variation in E[s] and *p*, then we conclude that the efficacy of selection against modifiers of $\mu_{E}$ is determined by $N_{e}$ alone, while the efficacy of selection against modifiers of $\mu_{O}+\mu_{S}$ is determined by the product $gN_{e}$. Fig. 4*B* summarizes how *g* and $N_{e}$ interact to shape the gamete mutation load.

**Fig. 5.**
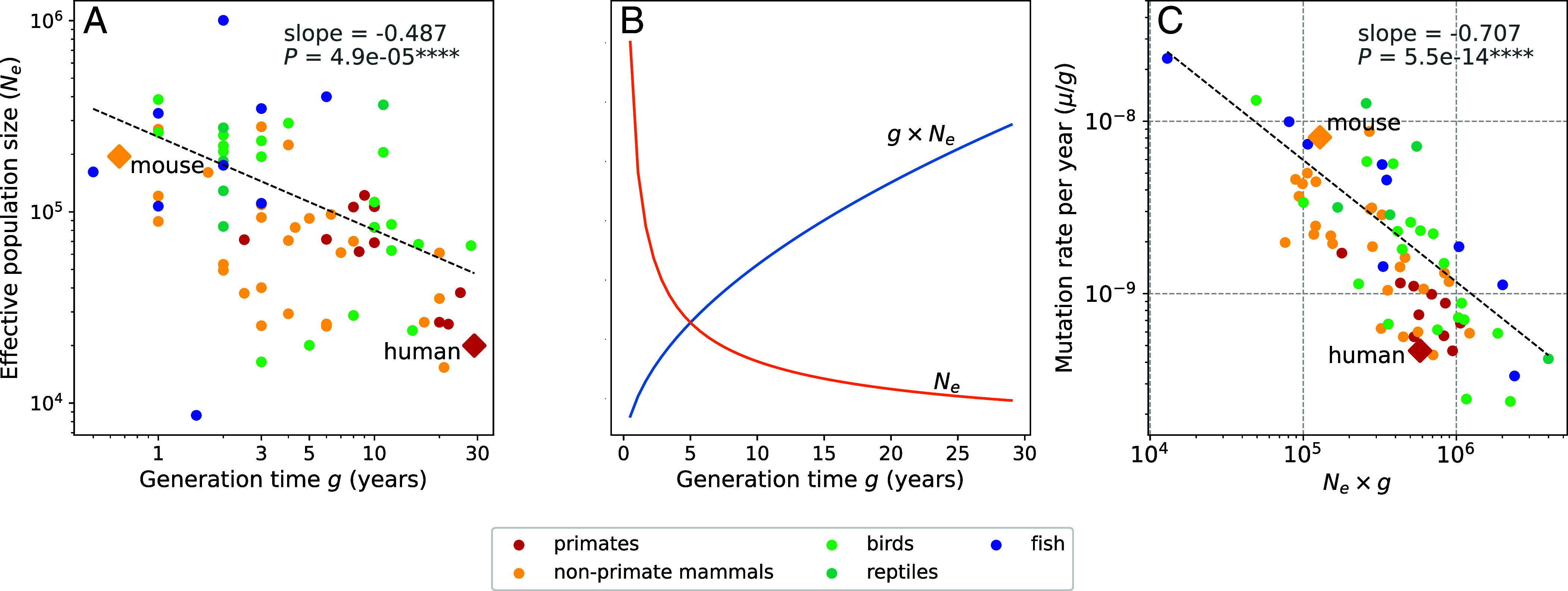
The relationship among *N_e_*, generation time, and the strength of selection against clocklike mutator alleles. (*A*) The parameters log(*N_e_*) and log(*g*) are inversely correlated in the Wang and Obbard mutation rate data (26). We estimate a slope of −0.487 based on a PGLS regression. (*B*) Expected values of $N_{e}$ and ${\mathrm{gN}}_{e}$ as functions of *g,* extrapolated from the regression line in panel *A* and converted from log scale to natural scale. Each curve has been visualized using an arbitrary *y*-axis scaling, and together they illustrate that ${\mathrm{gN}}_{e}$ increases with increasing *g* even as $N_{e}$ decreases. (*C*) Mutation rate estimates from Bergeron et al. confirm that the mutation rate per year decreases as a function of ${\mathrm{gN}}_{e}$, as expected if long generation times dominate the effect of decreasing effective population size to strengthen selection against clocklike mutator alleles. Note that the long-lived primates have higher values of ${\mathrm{gN}}_{e}$ than the short-lived, high-$N_{e}$ mouse.

Our calculations suggest that the species with the lowest germ cell mutation rates will be the species for which $gN_{e}$ is the largest. However, the inverse correlation between *g* and $N_{e}$ means that it is not obvious which life history strategies will maximize $gN_{e}$. To gain clarity, we note that the relationship between $N_{e}$ and *g* was previously studied in some detail during the initial development of the nearly neutral theory, since it was needed to explain the consistency in molecular substitution rates across the tree of life (63, 64). In this context, Chao and Carr previously measured an inverse log–linear correlation between $N_{e}$ and *g* (60). We were able to reproduce this log–linear relationship in the Wang and Obbard mutation rate data (26) (Fig. 5*A*).

The linear relationship $\log\;N_{e}=\gamma \;\log\;g+\log\;C$ (where γ and *C* are constants) implies that $N_{e}=Cg^{\gamma }$ and ${\mathrm{gN}}_{e}=Cg^{1+\gamma }$. This expression might increase or decrease with increasing *g* depending on whether γ is greater or less than −1, so knowing the value of γ is key to deciding whether species with long or short generation times are likely to have the lowest mutation rates. We estimate that γ≈-0.487 based on a PGLS regression of log(*N_e_*) against log(*g*), remarkably close to the value of −0.5 that Kimura and Ohta originally proposed to reconcile the nearly neutral theory with the molecular clock model (60, 65). This implies that ${\mathrm{gN}}_{e}=g^{1+\gamma }=g^{1-0.487}=g^{0.513}$. As shown in Fig. 5*B*, this implies that ${\mathrm{gN}}_{e}$ behaves approximately like $\sqrt{g}$, increasing as *g* increases. Therefore, if we compare fast-reproducing species like mice to slower-reproducing species like humans, the slower-reproducing species will have smaller values of $N_{e}$ but larger values of ${\mathrm{gN}}_{e}$, which is the parameter that determines the strength of selection for a low mutation rate per year in the germ cells. Fig. 5*C* shows empirically that ${\mathrm{gN}}_{e}$ is negatively correlated with the germline mutation rate per year, consistent with the idea that the parameter $gN_{e}$ determines the strength of selection against mutator alleles. We can also see that humans and other long-lived primates have high values of ${\mathrm{gN}}_{e}$ compared to the short-lived mouse.

## Discussion

Variation in the germ cell mutation rate per year appears to echo patterns of mutation rate variation in somatic tissues. A recent study of colon crypt mutations found an inverse log–log linear relationship between lifespan and the mutation rate per year (56), mirroring the correlation we observe between generation time and the mutation rate in the gametes. In both cases, the fitness effect of any mutation rate increase becomes compounded over the lifetime of the cell lineage that is mutating, giving long-lived, late-reproducing organisms a stronger incentive to preserve genomic integrity (66, 67). In gerontology, this concept is known as the disposable soma theory (68, 69), and our analysis suggests that a version of this theory is also applicable to the germline. Since the same molecular machinery is ultimately responsible for safeguarding both germline and somatic DNA, pleiotropy between somatic and germline mutation rates may amplify differences among species in the strength of selection against clocklike mutator alleles. Our theoretical work underscores the value of collecting mutation rate data in a way that facilitates separate estimation of embryonic and germ cell mutation rates, whether by sequencing multioffspring pedigrees (46, 47, 70) or using emerging technologies such as single-cell gamete sequencing (71, 72). The latter approach could avoid the statistical interdependence between $\mu_{E}$ and $\mu_{O}+\mu_{S}$ that occurs when these parameters are estimated from the slope and intercept of the same regression.

While selection against nearly neutral mutator alleles is a parsimonious explanation for the observation that longer generation times are associated with higher rates of prepuberty mutations and lower rates of germ cell mutations, other explanations are possible. For example, factors such as higher metabolism or higher sperm production volume may contribute to the higher germ cell mutation rates observed in fast-reproducing organisms (70, 73). The contribution of such factors may become measurable as additional generation-time-calibrated mutation rate estimates become available.

Our results raise a fascinating question regarding the evolution of germline mutagenesis: If the low germ cell mutation rates in late-reproducing species are driven by selection to reduce DNA damage or improve the molecular efficacy of DNA repair, then why don’t these improvements translate to lower mutation loads prepuberty as well? It is possible that later-reproducing species have higher prepuberty mutation loads simply because their development takes longer and involves more cell divisions, allowing more time for mutations to accumulate before the onset of reproduction. Drost and Lee previously argued that most mammals, including mice and humans, have similar primordial germ cell developmental trajectories, with similar numbers of cell divisions leading from the zygote to the germ cells (32). This implies that variation among mammals in the rate of prepuberty mutations is not likely driven by variation in the number of early embryonic cell divisions, but must be driven by other factors such as the length of time elapsed during embryonic development and the rest of prepuberty development. In principle, slower development could also leave more time for DNA repair, but our results suggest that this extra time does not keep slower-maturing species from accumulating more germline mutations before puberty than faster-maturing species.

One biological variable that we have not incorporated into our model is the distinction between domesticated and wild animals. Bergeron et al. (27) previously found that domesticated animals tend to have higher per-year mutation rates than wild animals, which might be due to their smaller effective population sizes or to selection for rapid reproduction in captivity. In particular, laboratory mice have a long history of domestication that might contribute to their high mutation rates. An important future direction will be to measure mutation rates from more wild animals with generation times less than 1 y, which will help determine whether laboratory mice are an outlier or a representative model of mutagenesis in a fast-reproducing rodent. However, laboratory mice are not an obvious outlier in Figs. 2 or 3 regressions, but instead have a similar mutation rate to wild species with similar generation times that were sequenced by Bergeron et al. (27).

Although our results do not indicate that laboratory mice have unusual germline mutation rates, we did infer that aye-ayes have an unusually low prepuberty mutation load $\mu_{E}$. Wang et al. previously reported an unusual pattern of female mutation bias in the offspring of aye-aye mothers older than 15 y at conception, and we used multiple datasets to infer that the rate of oocyte mutations is consistently higher than the rate of spermatogonial mutations throughout the aye-aye reproductive lifespan. The case study of the aye-aye suggests that the relationship of $\mu_{E}$ and $\mu_{O}+\mu_{S}$ to generation time is highly informative about which species’ mutational processes deviate from the norm.

In theory, $\mu_{E}$ and $\mu_{O}+\mu_{S}$ should exhibit less within-species variation compared to mutation rate estimates that depend on the age range of the trio parents represented in the dataset. However, we also found substantial batch effects when we inferred $\mu_{E}$ and $\mu_{O}+\mu_{S}$ from different studies of de novo mutation in the same species. These inconsistencies are likely driven by differences in bioinformatic pipelines for inferring DNM rates, which are still an active area of technical innovation and thus quite variable between studies. Since no existing DNM calling pipelines have been explicitly designed to estimate the parameters $\mu_{E}$ and $\mu_{O}+\mu_{S}$ as accurately as possible, further technical work may be needed to refine such estimates and gain a more complete understanding of how they vary among species.

In addition to making testable predictions about the molecular efficacy of DNA repair and how it varies among species, our model provides a straightforward way to impute the germline mutation rates of species for which direct measurements are missing. If a species’ age of reproductive maturity and average generation time have both been estimated, Eq. **9** provides a mutation rate estimate that can be used for calibrating phylogenetic trees and demographic histories. Although such a mutation rate estimate will not be as accurate as a mutation rate estimated directly from trio sequencing data, it may be more reliable than attempting to infer the mutation rate from phylogenetic data (71, 72), which famously overestimated the human mutation rate by a factor of 2 (74–76) and also reached inaccurate conclusions about baleen whale mutation rates (77). Our model may even be useful for imputing the mutation rates of nonmammalian species; for example, the mutation rate of the black abalone is similar to the mutation rates of vertebrates with similar reproductive lifespans (78). We have not attempted here to deduce how mutation rates are affected by body size (73), domestication history (79), or the countless other variables that may affect genomic integrity, but a good model encapsulating the effects of generation time should improve our power to learn the effects of additional variables in the future.

## Methods

### Ascertainment of De Novo Mutation Counts from Published Trio Data.

#### Human.

Our human mutation parameter estimates are derived from Jonsson et al. (38) and Wu (41). For the Jonsson data, the number of mutations and the parental ages for each trio were obtained from ref. 38. A multiplicative correction factor of 1.009, which adjusts for the original paper’s estimated number of false positives and negatives, was applied to the number of mutations identified in each trio. The haploid callable genome size was 2,682,890,000 bp. Both values were found in the *Methods*. For the Wu data, parental age was obtained from S2 Table (41). Mutation count was obtained from S2 Data (41), under the sheet name “Figure 2*A*,” and callable haploid genome size was 2,881,033,286, as reported in the *Methods*. The two datasets were combined and no significant batch effect was found, as we describe in “testing for batch effects in species with multiple mutation rate datasets” (Dataset S3).

#### Chimpanzee.

Mutation data were obtained from Besenbacher et al. (53). Parental age, mutation counts, callable base pairs were all obtained from Table 1 (53). Note that we did not attempt to combine these with mutation counts from Venn et al. (39) because the same trios were used in the two studies.

#### Olive baboon.

Mutation data were obtained from Wu et al. (41) and Wang et al. (52). For the Wu data, parental age was obtained from S3 Table (41). Mutation counts were obtained from S2 Data (41), under the sheet named “Figure 2*B*,” and callable haploid genome size was 2,581,196,250, as reported in the *Methods*. For the Wang data, mutation counts, parental ages, and callable genome sizes were all obtained from Table 2 (52).

#### Rhesus macaque.

Mutation data were obtained from Wang et al. (42) and Bergeron et al. (43). For the Wang data, parental ages, mutation counts, callable genome sizes were all obtained from Table 1 in the main text (42), and in Supplementary Table S1 for the Bergeron data (43). The two datasets were combined, and no significant batch effect was found (Dataset S3).

#### Owl monkey.

Mutation data were obtained from Thomas et al. (40). Parental ages and mutation counts were obtained from the supplementary spreadsheet, under the sheet name “DataS1C-rates” (40). Callable genome size was calculated as 2×(Total sites-Filtered SNP sites), both of which were also sourced from the same table. A false negative rate (FNR) of 0.4376 was applied to correct for the mutation count by dividing the raw count by (1-FNR). Since Thomas et al. report paternal and maternal generation times of 6.64 and 6.53, we use an owl monkey generation time of 6.6 y.

#### Domestic cat.

Mutation data were obtained from Wang et al. (44). Parental age, mutation count were obtained from Table 1 in the main text. Callable genome size was calculated as 2×Haploid size×Callability, both of which were also sourced from Table 1 (44).

#### Mouse.

We downloaded the supplementary mutation data from Lindsay et al. (49), which reports accessible-genome-corrected mutation counts and parental age at conception in weeks for all of the offspring in their pedigrees. Haploid genome size was reported to be 2,222,635,788 bp in the *Methods*.

#### Aye-aye.

Mutations were obtained from Versoza et al. (51) and Wang et al. (52). For the Versoza data, parental ages and mutation counts were obtained from Supplementary Tables 1 and 3 respectively, and the callable haploid genome size was estimated to be 2,279,228,391 bp, as reported in Supplementary Table 2 (51). For the Wang data, mutation counts, parental ages, and callable genome sizes were all obtained from Table 1 (52).

#### Testing for batch effects in species with multiple mutation rate datasets.

There exist multiple independent trio datasets for humans and rhesus macaques. In order to test for systematic differences in the mutation rates estimated from each dataset, we performed an additional regression for each species that included the dataset source as a categorical variable and allowed for interaction between the dataset category and both the coefficients $\mu_{E}$ and $\mu_{O}+\mu_{S}$. An ANOVA test was performed to check for the significance of the interactions, and no batch effect was detected in either species.

### Estimating the Relative Rates of Spermatogonial and Oocyte Mutations.

For each of the above species, we sought to estimate the number of germline mutations that accumulate per year of parental age. This requires averaging together each trio’s maternal and paternal ages in proportion to the relative rates of mutations occurring in the spermatogonia versus the oocytes postpuberty, as motivated by Eqs. **2**–**4**. To this end, we estimated the proportion of spermatogonial mutations $\mu_{S}/\left(\mu_{O}+\mu_{S}\right)$ using a likelihood function derived by Gao et al. [“*Estimating Sex-specific Mutation Parameters with a Model-based Approach*” from the *Methods* of (50)]. To reproduce this likelihood function here for completeness, we will need some additional notation: First, we will decompose the mutation rate parameter $\mu_{E}$ into $\mu_{E,M}$ (the component of $\mu_{E}$ arising on the maternal chromosome) and $\mu_{E,P}$ (the component of $\mu_{E}$ arising on the paternal chromosome). For each trio in a given dataset, indexed by *i*, we will let ${A_{M}}^{i}$ and ${A_{P}}^{i}$ denote the maternal and paternal age displacements from puberty (i.e., the maternal and paternal ages minus the age of puberty *P*). Finally, we will let ${U_{M}}^{i},{U_{P}}^{i}$, and ${U_{X}}^{i}$ denote the numbers of maternally phased, paternally phased, and unphased mutations observed in trio *i*. In terms of the mutation rate parameters $\mu_{E,M}{,\mu }_{E,P}{,\mu }_{S}$, and $\mu_{O}$, Gao et al. showed that the log likelihood of the observed mutations is equal to the following function *L* plus a constant:

[17] $$\begin{array}{c} L=\sum_{i=1}U_{M}^{i}\text{log}\left(\mu_{E,M}+\mu_{O}A_{M}^{i}\right)+U_{P}^{i}\text{log}\left(\mu_{E,P}+\mu_{S}A_{P}^{i}\right)+U_{X}^{i}\text{log}(\mu_{E,M}+\mu_{O}A_{M}^{i}\\ \;+\mu_{E,P}+\mu_{S}A_{P}^{i})-(\mu_{E,M}+\mu_{O}A_{M}^{i}+\mu_{E,P}+\mu_{S}A_{P}^{i}). \end{array}$$

We maximized this log likelihood function using the BFGS algorithm in R with the package bbmle and then used these maximum likelihood parameters to calculate species-specific values of $\mu_{S}/\left(\mu_{O}+\mu_{S}\right)$ for substitution into Eq. **3**.

Given the small number of phased mutations available for these species, we also devised an alternative approach where we let $\mu_{S}/\left(\mu_{O}+\mu_{S}\right)$ vary across a grid of values between 0 and 1. For each of these values, we computed McFadden’s Pseudo-$R^{2}$ (${R_{\mathrm{McF}}}^{2}$) of the Poisson regression (Eq. **4**) and identified the value that maximized this correlation between average parental age and mutation rate. For most datasets, these two approaches yielded similar results–the $\mu_{S}/\left(\mu_{O}+\mu_{S}\right)$ that maximized ${R_{\mathrm{McF}}}^{2}$ was close to the value that maximized the likelihood in Eq. **17**. In the Versoza aye-aye dataset, which had very few phased mutations, we found that ${R_{\mathrm{McF}}}^{2}$ was maximized at $\mu_{S}/\left(\mu_{O}+\mu_{S}\right)=0$, suggesting a strong maternal contribution in concordance with our inference from the Wang et al. dataset (52).

### Measurement of the Correlation Between Mutation Rate Per Year and Generation Time Across Vertebrates.

We used the nucleotide diversity (π), mutation rate per generation (μ) and generation time (g) data compiled by Wang and Obbard to quantify the relationship between *g* and $N_{e}$. We calculated each species’ mutation rate per year ($\mu_{y}$) by dividing μ by g. We first estimated $N_{e}$ for each species via the formula $N_{e}=\pi /\left(4\cdot \mu \right)$ (26) (*Data, Materials, and Software Availability*). We then performed a PGLS regression of the per-year mutation rate $\mu_{y}$ against ${g\cdot N}_{e}$ using the R library caper (80). Additionally, we estimated Pagel’s λ (81) to be 0.92 using caper’s maximum likelihood implementation. λ is commonly used to quantify the amount of phylogenetic signal in the dataset. It is a scaling parameter applied to internal branch lengths in the phylogenetic tree, and is typically a value between 0 and 1. λ=1 means that the traits being regressed against one another appear to have evolved according to a Brownian motion evolutionary model and is interpreted as strong evidence for phylogenetic signal in the dataset, whereas λ=0 suggests that the traits evolved completely independently of the phylogenetic tree structure. See Dataset S6 for detailed numerical regression results.

## Supplementary Material

Appendix 01 (PDF)

Dataset S01 (XLSX)

Dataset S02 (XLSX)

Dataset S03 (XLSX)

Dataset S04 (XLSX)

Dataset S05 (XLSX)

Dataset S06 (XLSX)

## Data Availability

The mutation rates, nucleotide diversity, generation time data, and phylogenetic tree utilized in our calculations were originally compiled by Wang and Obbard (26) and are all publicly available at ref. 82. The code we used to perform this paper’s analysis is available at ref. 83.
