# Supplementary material for: Population size interacts with reproductive longevity to shape the germline mutation rate
Source: Proc Natl Acad Sci U S A. 2025 May 20;122(21):e2423311122. doi: 10.1073/pnas.2423311122 (PMC12130860; doi:10.1073/pnas.2423311122)
Supplement: Supplementary file 1 — Appendix 01 (PDF) [file pnas.2423311122.sapp.pdf]

Supplementary Figures

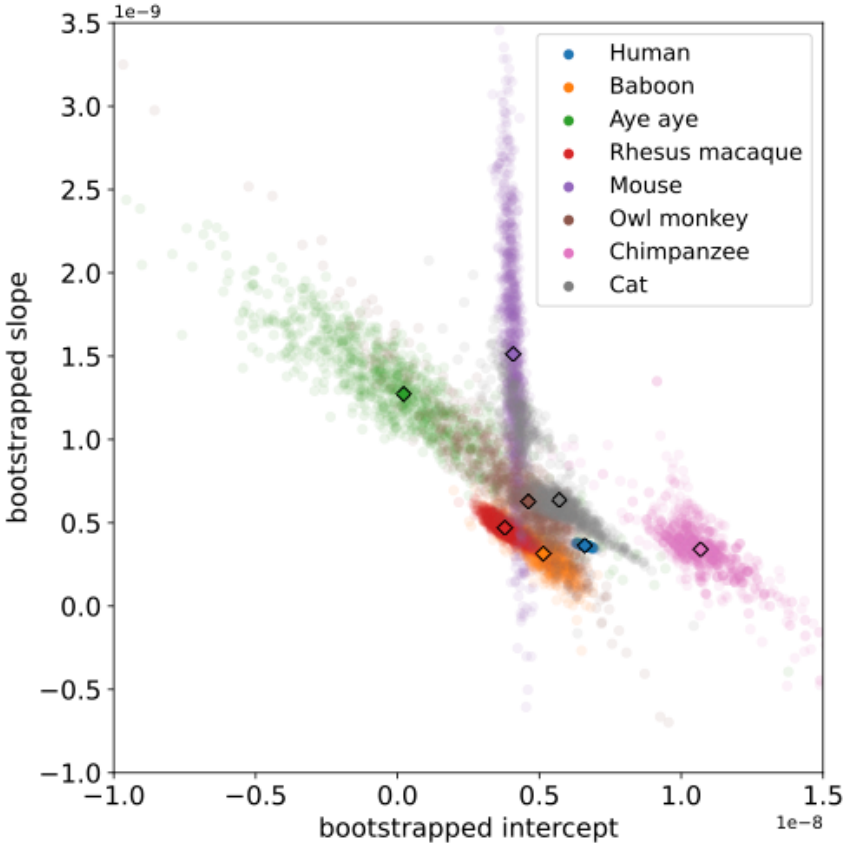

Supplementary Figure 1: Bootstrapped confidence intervals of mutation rate parameter estimates μ_E and $\mu_O + \mu_S$. For each species, we generated 1000 bootstrap replicates by resampling trios with replacement and then performing a PGLS regression on the resampled trios' mutation counts. The distribution of regression parameters μ_E and $\mu_O + \mu_S$ across replicates are shown for each species.

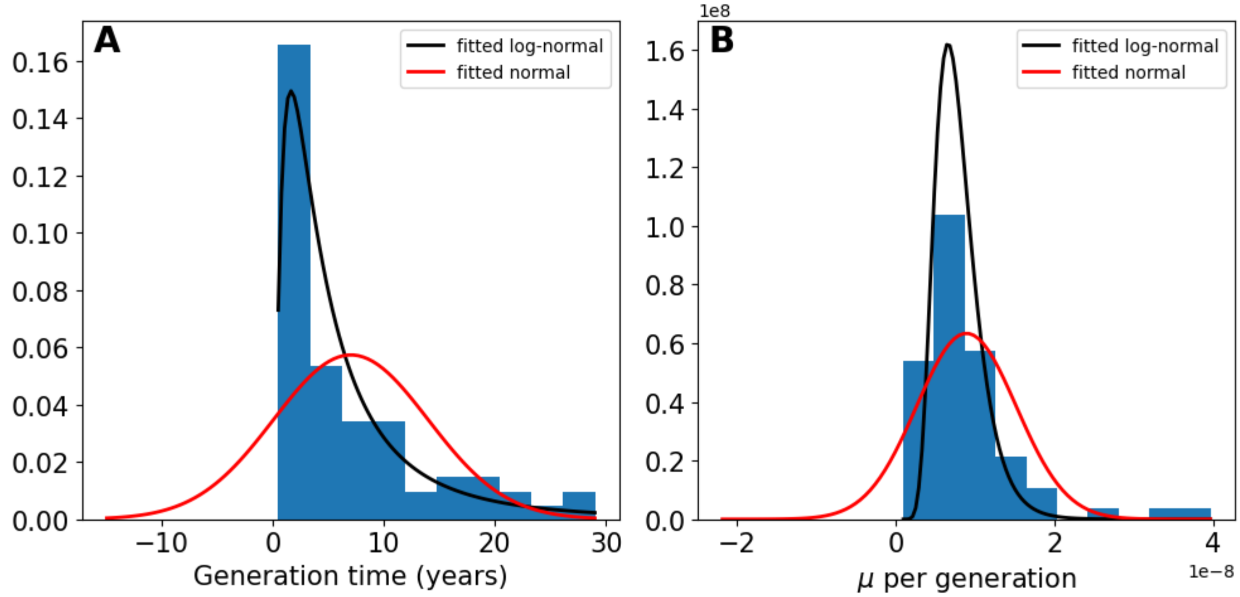

Supplementary Figure 2: Distributions of generation time and mutation rate per generation across species. Data taken from Wang and Obbard (26). Red and black lines correspond to the fitted normal and log-normal distributions, respectively. Lognormal provides a better fit to both the distribution of generation times and the distribution of the mutation rate per generation. The unit of the y-scale corresponds to the mutation count in each histogram bin.

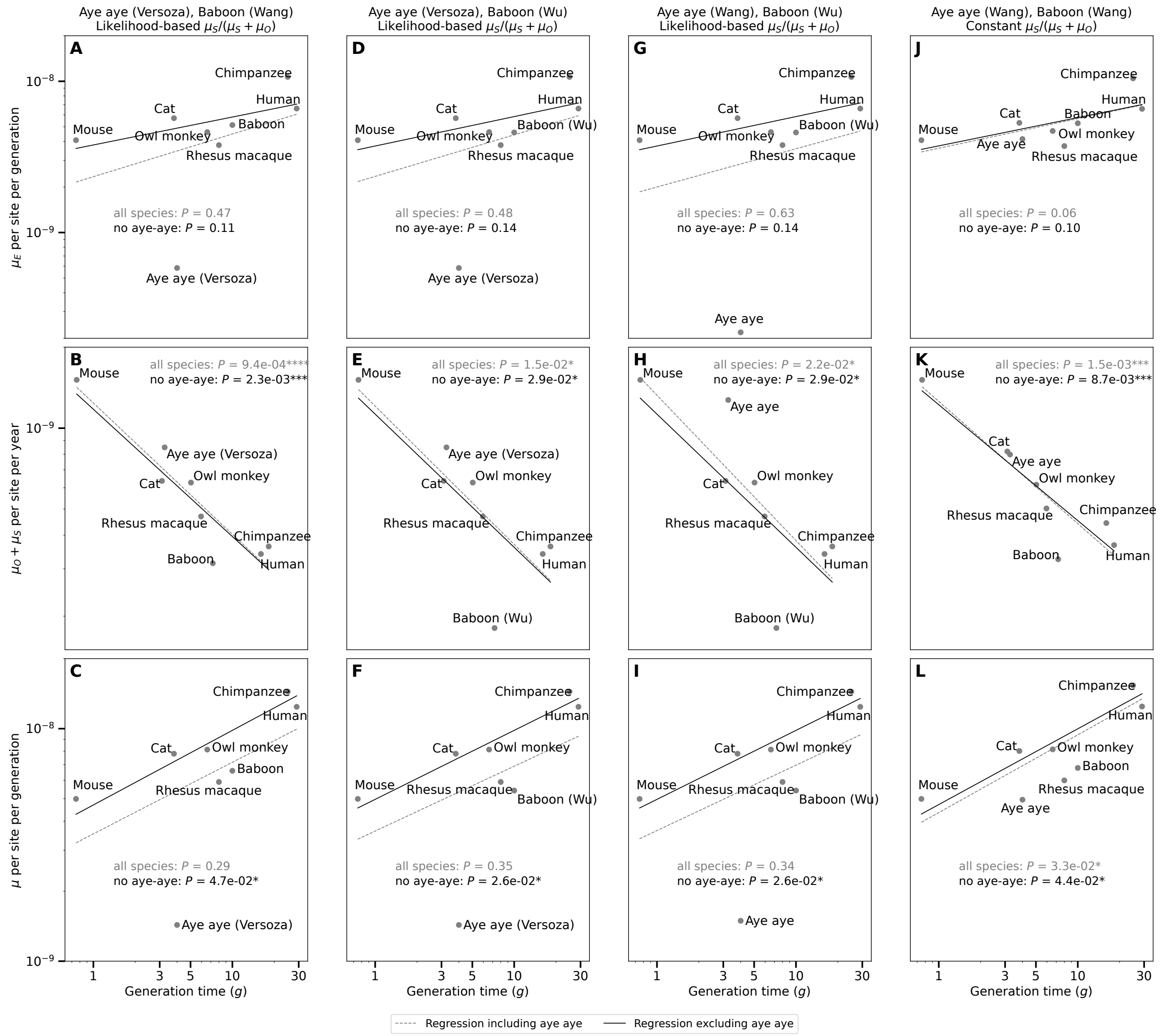

Supplementary Figure 3: PGLS regressions of μ_E and $\mu_O + \mu_S$ versus generation time using alternate datasets and an alternate scheme for computing the average parent generation time. Each column of panels shows regressions of μ_E , $\mu_O + \mu_S$, and μ that were estimated using an alternative set of data. Panels **A–C** utilize alternative aye-aye mutation calls that were generated by Versoza, et al. (51) rather than the Wang, et al. paper (52) used to generate these datapoints for **Figure 2.** Panels **D–F** use the Versoza, et al. aye-aye data (51) as well as an alternative baboon dataset generated by Wu, et al. (41). Panels **G–I** use only the alternative Wu, et al. baboon data. Finally, Panels **J–L** use all of the same datasets used in the main text but average together paternal and maternal age in a 3-to-1 ratio α rather than the species-specific α estimates used in the main text analysis.

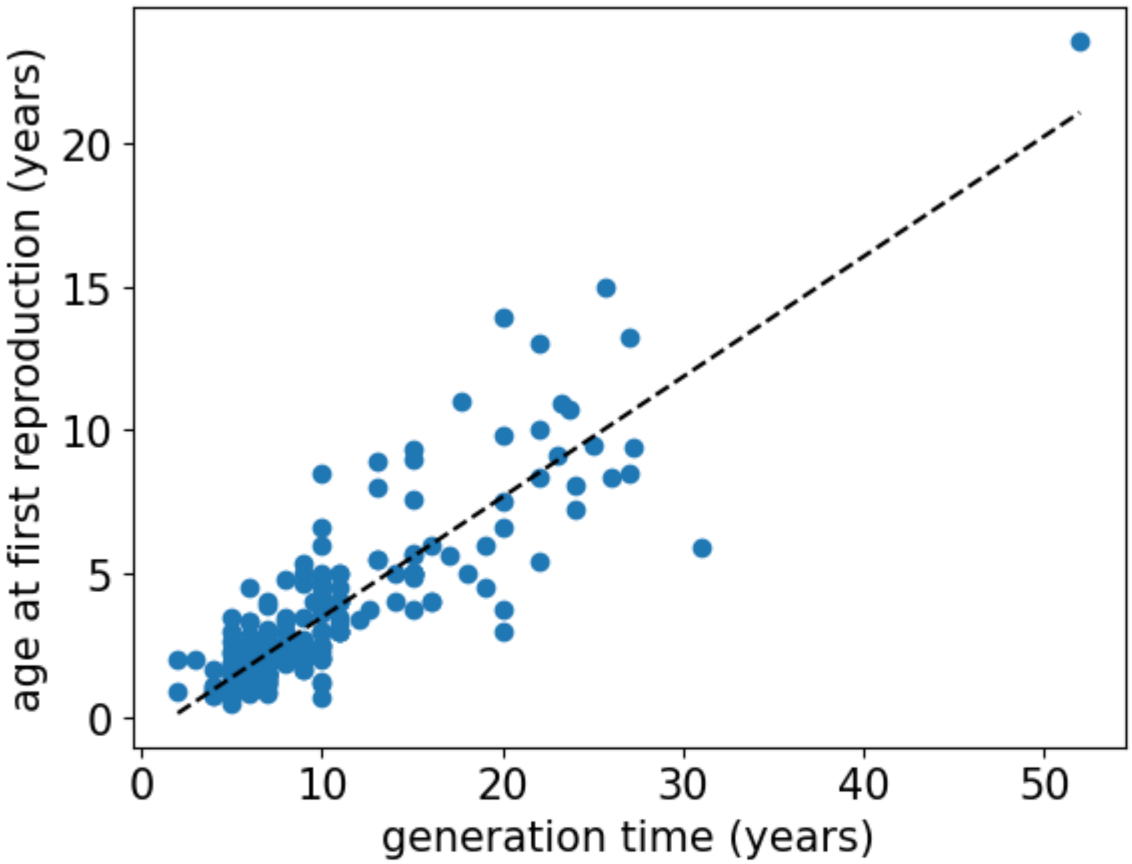

Supplementary Figure 4: Regression of age at first reproduction versus generation time. Data taken from Pacifici et al. (57). Age at first reproduction is used as a proxy for age at puberty. Age at first reproduction is found to be linear with respect to generation time, with a slope of 0.42.
